# Air, surface, and wastewater surveillance of SARS-CoV-2; a multimodal evaluation of COVID-19 detection in a built environment

**DOI:** 10.1038/s41370-025-00757-3

**Published:** 2025-03-01

**Authors:** Andreas Olsen Martinez, Leslie G. Dietz, Hooman Parhizkar, Devrim Kaya, Dale Northcutt, Patrick F. Horve, Jason Stenson, Michael Harry, David Mickle, Shana Jaaf, Oumaima Hachimi, Casey Kanalos, Isaac Martinotti, Garis Bowles, Mark Fretz, Christine Kelly, Tyler S. Radniecki, Kevin Van Den Wymelenberg

**Affiliations:** 1 Biology and the Built Environment Center, University of Oregon, Eugene, OR 97403 USA; 2 Institute for Health and the Built Environment, University of Oregon, Portland, OR 97209 USA; 3 Los Alamos National Laboratory, Los Alamos, NM 87544 USA; 4 School of Chemical, Biological, and Environmental Engineering, Oregon State University, Corvallis, OR 97331 USA; 5 Energy Studies in Buildings Laboratory, University of Oregon, Eugene, OR 97403 USA; 6 Environmental and Occupational Health Sciences Institutes (EOHSI), Rutgers University, Piscatawy, NJ 08854 USA; 7 School of Public Health and Imperial Valley, San Diego State University, San Diego, CA 92182 USA; 8 Institute of Molecular Biology, University of Oregon, Eugene, OR 97403 USA; 9 College of Architecture, University of Nebraska, Lincoln, NE 68588 USA

**Keywords:** Surveillance, Built Environment, COVID-19, SARS-CoV-2, Ventilation, Wastewater

## Abstract

**Background:**

Environmental surveillance of infectious organisms holds tremendous promise to reduce human-to-human transmission in indoor spaces through early detection.

**Objective:**

In this study we determined the applicability and limitations of wastewater, indoor high-touch surfaces, in-room air, and rooftop exhaust air sampling methods for detecting SARS-CoV-2 in a real world building occupied by residents recently diagnosed with COVID-19.

**Methods:**

We concurrently examined the results of three 24-hour environmental surveillance techniques, indoor surface sampling, exhaust air sampling and wastewater surveillance, to the known daily census fluctuations in a COVID-19 isolation dormitory. Additionally, we assessed the ability of aerosol samplers placed in the large volume lobby to detect SARS-CoV-2 multiple times per day.

**Results:**

Our research reveals an increase in the number of individuals confirmed positive with COVID-19 as well as their estimated human viral load to be associated with statistically significant increases in viral loads detected in rooftop exhaust aerosol samples (*p* = 0.0413), wastewater samples (*p* = 0.0323,), and indoor high-touch surfaces (*p* < 0.001)). We also report that the viral load detected in lobby aerosol samples was statistically higher in samples collected during presence of occupants whose COVID-19 diagnostic tests were confirmed positive via qPCR compared to periods when the lobby was occupied by either contact-traced (suspected positive) individuals or during unoccupied periods (*p* = 0.0314 and <2e−16).

**Significance:**

We conclude that each daily (24h) surveillance method, rooftop exhaust air, indoor high-touch surfaces, and wastewater, provide useful detection signals for building owner/operator(s). Furthermore, we demonstrate that exhaust air sampling can provide spatially resolved signals based upon ventilation exhaust zones. Additionally, we find that indoor lobby air sampling can provide temporally resolved signals useful during short duration sampling periods (*e.g.,* 2-4 hours) even with intermittent occupancy by occupants diagnosed with COVID-19.

**Impact:**

- Our research demonstrates that aerosol sampling can detect COVID-19 positive individuals in a real world lobby setting during very short occupancy periods. We demonstrate the effectiveness of rooftop exhaust aerosol, surface, and wastewater environmental surveillance in monitoring viral load in building occupants, both at the building scale and with ventilation zone-level resolution for aerosols. We provide actionable data for researchers, health officials and building managers who seek to determine which monitoring method is best for their building or study. This study is relevant in the fields of epidemiology, exposure sciences, biomonitoring, virology, public health, and healthy building design and management.

## Introduction

Towards the end of 2019, severe acute respiratory syndrome coronavirus 2 (SARS-CoV-2), a highly transmittable airborne virus, spread across the globe causing coronavirus disease 2019 (COVID-19), triggering a global pandemic with devastating impacts to human lives, livelihoods and social networks within just a few months [1]. Individuals who contract COVID-19 can present a wide variety of symptoms, from asymptomatic, to mild, or severe symptoms which can persist for long periods of time, and can lead to hospitalization, a need for long-term care, and death [2–5].

As the virus continues to circulate and infect new individuals, new variants are being identified [6, 7]. There has been extensive documentation of outbreaks related to SARS-CoV-2 transmission in built environments indicating that indoor environments, including work spaces, concert halls, multi-family dwellings, long-term care facilities, fitness gyms, prisons, schools, restaurants, cruise ships, airlines and places of worship, are among the primary environments for COVID-19 spread via the airborne route [8–27]. As such, environmental mitigation strategies including ventilation, high-efficiency filtration, and humidification have been employed to save lives, reduce disease transmission, and limit the emergence of new variants of concern (VOC) [28–30].

Due to the potential for viral shedding and contagiousness prior to symptom onset, researchers and public health officials have explored SARS-CoV-2 environmental surveillance as a method of early detection and outbreak prevention [19, 31–33]. Available types of pathogenic environmental surveillance include air sampling, surface sampling, and wastewater sampling [30, 33–35]. These surveillance techniques have been utilized with increasing frequency since the beginning of the COVID-19 pandemic to better guide cleaning practices, detect and quantify environmental viral loads associated with COVID-19 infections, reduce outbreaks, and track community spread [15, 16, 31, 35–38]. In addition to well-documented wastewater sampling programs [38–41], SARS-CoV-2 genetic material has been found in HVAC filters, exhaust air duct screens and rooftop air-handling equipment in buildings with individuals diagnosed with COVID-19, suggesting the value of building (often roof-top) exhaust air as a sampling location for surveillance [13, 42, 43]. Many studies deployed air sampling methods in close proximity to patients diagnosed with COVID-19 for short sampling periods; however, effectiveness of these sampling methods during longer sampling periods and with far-field placement (i.e., locating air sampling within building exhaust outlets or in large communal spaces) are less characterized [15, 30, 32, 34, 37].

**Fig. 1:**
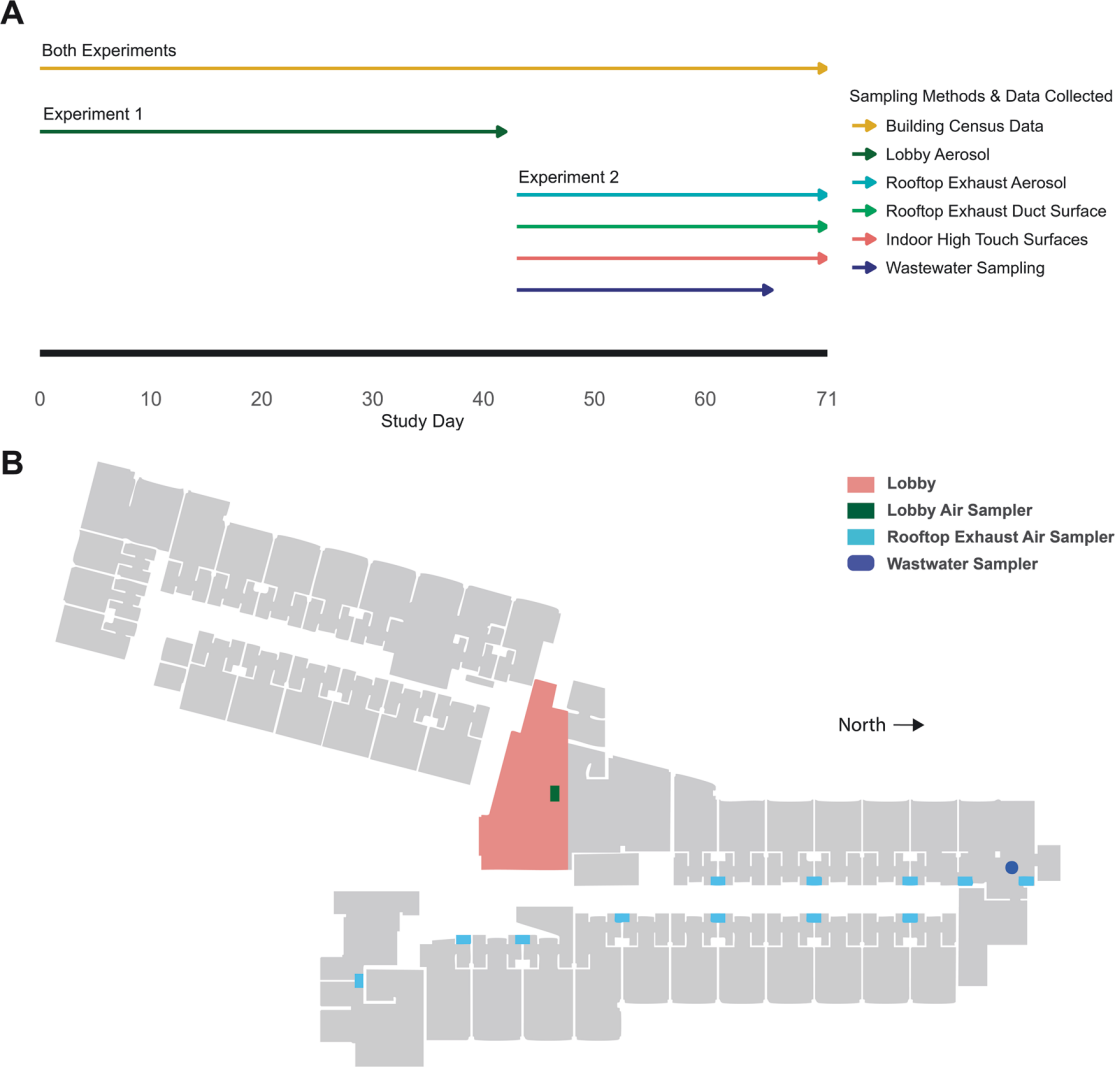
Timeline and sampling locations for experiments 1 and 2. Figure **A** is the study timeline depicting when Experiment 1 and Experiment 2 occurred in relation to each other and what types of data/sampling comprised each experiment, census data included number or occupants and each occupants lab test Cycle Threshold (C_T_) score. Figure **B** is the top-down view of isolation dormitory sampling locations. With the lobby represented in orange and the sampler locations shown in green, cyan and blue. Rooftop exhaust air samplers and rooftop duct samples are on the roof, all other samplers are on the ground floor. All occupants discussed in Experiment 2 were placed on the north wing (bottom of image), no samples were collected from the south wing of the building (top of image).

In this study, we conducted two primary experiments: Experiment 1 explored the effectiveness of air sampling for detecting SARS-CoV-2 in a large volume lobby space at a COVID-19 quarantine dormitory for specific time periods each day for a duration of 43 consecutive days (Fig. 1A). The lobby was occupied following a specific daily schedule by individuals A) presumed to be, or B) confirmed to be positive with COVID-19, while at other times C) the lobby was unoccupied, and our sampling time periods matched this schedule. Experiment 2 evaluated the surveillance effectiveness across the north wing of the same quarantine dormitory when concurrently sampling from 1) building rooftop exhaust air, 2) from several indoor common area surfaces, and 3) from wastewater, all using a 24-h sample collection period for the duration of several weeks (Fig. 1A) and capturing a range of occupant densities.

## Methods

### Study location

**Fig. 2:**
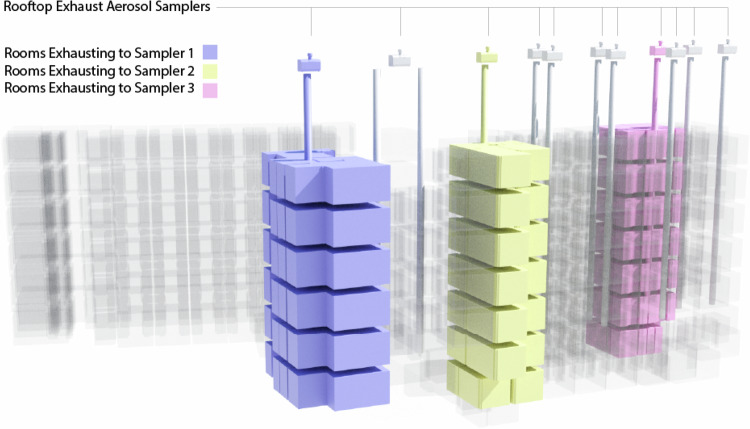
A depiction of how dormitory rooms are vertically grouped and where AerosolSense samplers collected from rooftop exhaust air stacks. The three highlighted room stacks show sampler layouts, paired dorm rooms, and indicate how rooms grouped around a common exhaust air stack with the AerosolSense air samplers on the roof. In total, 12 exhaust air stacks were monitored, encompassing air from 141 dorm rooms. This figure supports experiment 2.

At the time of study, the University of Oregon required all students living in campus dormitories to undergo weekly SARS-CoV-2 qPCR testing to determine COVID-19 status. Students who tested positive for COVID-19 or had been contact-traced to someone that was confirmed to be positive with COVID-19 were required to isolate for a 10–14-day period at the University of Oregon’s designated quarantine and isolation dormitory (Fig. 1B). The isolation dormitory was a seven story 125,020 square-foot building with a north and south wing, one communal entrance lobby and a cafeteria (Fig. 2). Students were required to remain inside their dormitory rooms at all times, which included a full bathroom. The only permittance outside of quarantine rooms was a strictly scheduled, two-hour outdoor recreation period each day. Students confirmed positive with COVID-19 had outdoor time from 09:00 to 11:00 and students who had been contact-traced to COVID-19 exposure had outdoor time from 13:00 to 15:00 PM each day.

### Study timeline

Experiment 1 consisted of lobby aerosol sampling, began on 2/8/2021 (sampling day 1) and ran until 3/22/2021 (sampling day 42). Experiment 2, consisted of rooftop aerosol, indoor high touch surfaces, and wastewater sampling and began on 3/23/2021 (sampling day 43) and ran 28 days until 4/20/2021 (sampling day 71). Both experiments are depicted in Fig. 1.

### Air sampling in Experiment 1 and Experiment 2

The AerosolSense^TM^ 2900 (Thermo Scientific, Waltham Massachusetts, USA, Catalog #121561) is an active air sampler that draws air at a rate of 200 liters per minute (L/m) and collects biological material by impacting air onto AerosolSense Capture Media^TM^ (ACM) stored in the device. The ACM was collected after each sampling period (ranging from 2 to 13 h in Experiment 1, and 24 h in Experiment 2) and placed in a 5 mL centrifuge tube (Ibis Scientific, Las Vegas Nevada, USA, catalog #HWK5.0) containing 1 mL of DNA/RNA Shield (Zymo Research, Irvine California, USA, # R1100-250) using sterile forceps. All air and surface samples were transported to an on-campus BSL-2 laboratory for processing, analysis, and storage at the University of Oregon. Details regarding air sample processing and laboratory analysis are provided in Supplementary Information Section 1.1.

### Wastewater in Experiment 2

A liquid autosampler (ISCO, Lincoln Nebraska, USA, model 3700C) sampling strainer was inserted into the north cleanout pipe. The autosampler was set to extract 100 mL every 10 min for a 24-h period with the collected samples being kept on ice. Wastewater sample amounts fell within the 50 mL–10 L range, often well above the 50 mL required for analysis. Sample amounts greater than 50 mL were well mixed prior to aliquoting 50 mL for analyses. Samples were stored on ice and transported to Oregon State University (Corvallis, Oregon) where reverse transcriptase droplet digital polymerase chain reaction (RT-ddPCR) analysis was conducted. Details regarding water sample processing and laboratory analysis are provided in Supplementary Information Section 1.1.

### Human census and viral abundance data in Experiment 1 and Experiment 2

**Fig. 3:**
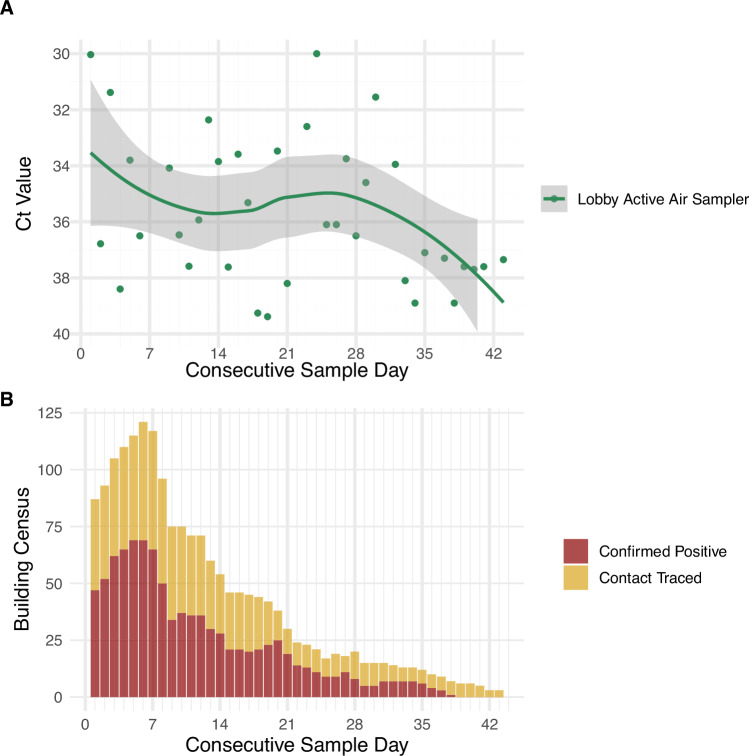
Comparing the lobby average detected viral load to daily the daily building census. Average daily C_T_ values of active air samplers placed in the lobby of the isolation dormitory (**A**) compared to the daily census counts for the entire quarantine building (**B**).The gray bar in Fig. Panel **A** represents the 0.95 confidence interval. Note that lower C_T_ values indicate higher viral abundance and higher C_T_ values indicate lower viral abundance. This figure supports Experiment 1.

Experiment 1 began on study day 1 and continued until study day 42. During the 43-day Experiment 1 study period, the isolation dormitory had a total population ranging from 3 to 121 (median 24) occupants, including occupants diagnosed with COVID-19 by qPCR test and contact-traced occupants who had been contact-traced to exposure events but had tested negative for COVID-19 via qPCR or who were awaiting test results. The population of confirmed positive occupants in the building ranged from 0 to 62 occupants during Experiment 1. On the first day of the 43-day study period, both the total population and the group of confirmed positive occupants started moderately high (confirmed positive = 47, building total = 87), peaked within the first seven days (confirmed positive = 69, building total = 121), and then slowly declined until the end of Experiment 1 (confirmed positive = 0, building total = 3, Fig. 3). Samples were collected on 39 days of the 43-day Experiment 1 study duration.

**Fig. 4:**
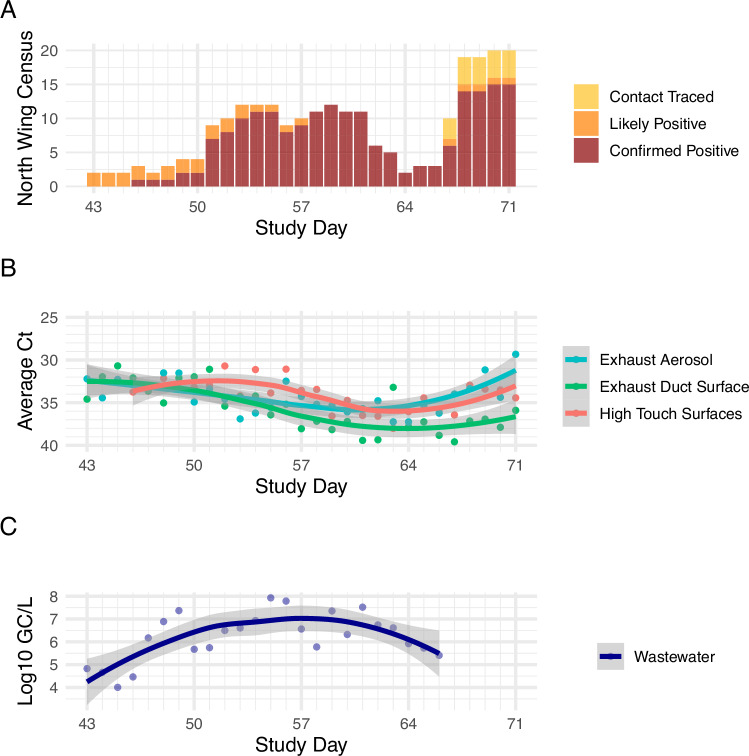
Comparing the daily North wing census to the average daily viral loads of multimodal samples. **A** Daily census data in the north wing of the isolation dormitory; **B** comparison of qPCR C_T_ values of rooftop exhaust aerosol samples, high-touch surfaces, exhaust duct surfaces and **C** wastewater logarithmic gene copies per liter data. Note that lower C_T_ values (panel **B**) indicate higher viral abundance and higher C_T_ values indicate lower viral abundance. This figure supports Experiment 2.

Experiment 2 began on study day 43 and the north wing begins with low occupancy (*n* = 2), rising to a mid range level of 10–12 occupants by day 53 and remains similar through day 61, dropping and then spiking again to a peak of 20 occupants on day 71 in the north wing during Experiment 2 (Fig. 4). Samples were collected on all 29 days of the 29-day Experiment 2 study duration.

De-identified and anonymized human census and COVID-19 qPCR viral abundance raw data were obtained from the University of Oregon Monitoring and Assessment Program (MAPs) through an honest broker data use agreement with the assistance of the University of Oregon Corporate Relations and Student Services and Enrollment Management departments. To better analyze environmental data within Experiment 2 the initial human viral load, specifically the qPCR viral abundance data that was used to diagnose the occupants for COVID-19, was used to calculate an estimated subsequent viral load per occupant by day (these methods are detailed in the “Estimating Human Viral Load Dynamics Over Isolation Period” section below).

#### Experiment 1 building lobby aerosol surveillance

Two AerosolSense^TM^ air samplers were deployed in the isolation dormitory lobby (710 square-feet). The samplers were placed in the center of the lobby at ground level, next to each other. All building occupants were required to enter and exit through this lobby using a prescribed schedule (Table 1). In addition, building staff and research staff passed through the lobby and all students and building staff were required to wear facemasks (any type), researchers were required to wear N-95 face masks. This experiment was conducted to determine if an aerosol sampler placed in a large air volume lobby is capable of detecting masked SARS-CoV-2 positive occupants as they move through the lobby for short periods of time. More information about the schedule (Table 1) is provided in the Supplementary Information Section 1.2.

**Table 1:** Lobby air sampling periods.

Time (24-h)	Lobby occupancy
09:00–11:00	Traffic from confirmed positive occupants (outdoor time)
11:00–13:00	Unoccupied (building and research staff only)
13:00–15:00	Traffic from contact-traced occupants (outdoor time)
15:00–17:00	Unoccupied (building and research staff only)
17:00–09:00	Unoccupied (building and research staff only)

Table 1 Note. Sample collection media was removed at the end of each sampling period. Staff and researchers periodically passed through the lobby between 0600 and 1900. Table 1 pertains to Experiment 1.

#### Experiment 2: comparing air, high touch surface and wastewater surveillance methods

For Experiment 2, rooftop exhaust air, rooftop exhaust duct surface, common area high-touch surfaces, and wastewater sampling methods were employed with a focus on the north wing of the building (Fig. 1B). The north wing was selected to compare the wastewater zone with a set of matching air and indoor surface zones. Additional information about north vs. south wing occupancy is provided in Supplementary Information Section 1.3).

Eleven rooftop exhaust stacks on the north wing were each fitted with a dedicated AerosolSense^TM^ air sampler. Each rooftop stack collects air from between 6 and 14 dormitory rooms. The groups of dorm rooms are organized in pairs per floor that share a common bathroom wall, and rooms stack vertically sharing a common air exhaust duct. Each room exhausts room air through that bathroom exhaust grille at a rate between 2800 and 8400 L/m (Fig. 1B, Fig. 2). These bathroom exhaust grilles are connected to a rooftop fan that operates continuously and serves as the building’s primary exhaust relief for the common area (lobby and hallway) supply ventilation system (Fig. 1B). The rooftop fans exhausted at an average rate of 5796.17 liters per minute(L/m). This rate remained fairly steady when the doors and windows to the dormitory room attached to each stack were closed but increased when the doors and windows were opened. Since occupant doors were required to be closed by the quarantine protocols, and the researchers observed windows were primarily kept closed by the occupants, the exhaust airflow was measured under these conditions. Individual rooftop fan exhaust rates and additional metrics and available in Supplementary Table 1. The building exhaust system is unfiltered. Rooftop exhaust air duct surface and rooftop exhaust aerosol sample viral load data were mapped to respective dormitory room groups and compared to room daily census occupancy data. ACM were collected from rooftop locations every 24-h period for 29 consecutive days.

Concurrent to rooftop exhaust air sampling, wastewater surveillance occurred at the north wing cleanout located in the dormitory maintenance shop (Fig. 2). To spatially relate the wastewater samples for direct comparison with the rooftop exhaust samples (12 rooftop exhausts), the north wing cleanout was selected as the wastewater sampling location. An endoscope (Forbest Products Co Model FB-PIC3188SD) was used to examine the sewer cleanout pipe conditions and identify ideal placement for the low-flow strainer. An ice-cooled composite ISCO 3700 autosampler (Teledyne ISCO, Lincoln, NE) was used to collect 90 mL of wastewater every 15 min for a 24-h period. The wastewater was collected daily at the same time and the autosampler was reset to collect on the same schedule to maintain sampling uniformity. To achieve sufficient and consistent sampling volumes, an inflatable IV bladder was inserted into the sewer cleanout pipe and was inflated to create a partial dam in the pipe where a small puddle of wastewater pooled. There is more information on the wastewater sampling setup in Supplementary Information Section 1.4 and wastewater sample laboratory analysis in Supplementary Information Section 1.1.

Concurrent to both wastewater and exhaust air sample collection, high-touch surfaces in the lobby and isolation floor were also sampled through surface swab techniques. Additional information about high-touch surface swabbing methods, including swabbing locations, is described in the Supplemental Information Section 1.4, sample processing and lab techniques are described in Supplemental Information.

### Statistical analysis

The statistical programming software environment R [41] was used to perform all statistical analyses. Air and surface samples were considered positive for SARS-CoV-2 if either the real time quantitative polymerase chain reaction (RT-qPCR) assay had a Cycle threshold (C_T)_) <=35. Wastewater samples were considered positive if the real time droplet digital polymerase chain reaction RT-ddPCR assay had three or more positive droplets. Trend comparisons between different methods of surveillance were computed using a plotted Locally Estimated Scatterplot Smoothing (LOESS) line with 95% confidence intervals. Pearson’s correlation coefficient was used to compare the average daily C_T_ values against the daily census counts for either the entire building census or the north wing census for Experiment 1 and Experiment 2 respectively. In Experiment 1, the different time segment sampling periods were compared using one-way analysis of variance (ANOVA) tests with the alternatives designated as the time periods closer to the most recent occupancy in the room by either confirmed positive or contact-traced students. ANOVA tests in Experiment 1 were performed on the log2 transformed C_T_ values to ensure that the data were normally distributed. In Experiment 2, viral abundance means for each sampling method were compared using two-tailed t-tests.

Significant changes in C_T_ values in rooftop aerosol samples and surface samples were studied through linear mixed models of the form $yi={X}_{i}\beta +{Z}_{i}{u}_{i}+\epsilon$ with the following descriptions: C_T_ value from aerosol samples and surface samples as response variable, census numbers associated with rooms connected to each rooftop exhaust stack as the fixed-effect term, and the interaction of estimated average daily human nasal C_T_ value and the cluster of rooms connected to each rooftop exhaust stack as the random-effect term.

Correlation between wastewater viral load and total number of confirmed positive occupants in north wing of the building was identified through the use of a generalized multi-linear model of the form $y={\beta }_{1}({x}_{1})+{\beta }_{2}({x}_{2})+{\ldots \beta }_{n}({x}_{n}+E)$ where *y* is the observed wastewater viral load, ${\beta }_{i}$ values are mixed linear regression coefficients for fixed effects values of building census as well as estimated average daily human nasal viral loads.

### Estimating human viral load dynamics over isolation period

Previous studies confirmed that there is a statistically significant positive correlation between the viral load detected in human samples and environmental viral load [30, 34]. To improve our assessment of the correlation between environmental sampling of viral loads and human census data described in this paper, we sought an estimation approach to incorporate the dynamics of human viral load over time as a random effect term. Researchers were given access to the results of RT-qPCR samples that were taken from each participant that was confirmed positive prior to their admission to the quarantine dorm. Using human C_t_ values collected prior to day one of the occupants’ dormitory isolation, as well as data from two clinical studies [34, 44], we estimated human viral decay over time for confirmed positive inhabitants. In one study, researchers characterized the kinetics of human viral loads and the presence or absence of viable SARS-CoV-2 in human samples from hospitalized COVID-19 patients [44]. In another study, we conducted a longitudinal analysis of human and environmental viral decay over time in patients isolated within a quarantine dormitory [34]. Data from both studies were used to derive a linear model that best explains human viral decay over time, resulting in surprisingly similar correlation models [34, 44]:

1 $${\rm{Y}}	=24.01977+0.67608\times {\rm{XY}}=24.01977+0.67608\times {\rm{XY}}\\ 	=24.01977+0.67608\times {\rm{X}}$$

2 $${\rm{Y}}	=23.8279+0.6663\times {\rm{XY}}=23.8279+0.6663\times {\rm{XY}}\\ 	=23.8279+0.6663\times {\rm{X}}$$

Where:

- **Y** represents the human viral load (or viral load in a specific sample type, such as nasal swabs or environmental samples) at a given time point.
- **X** represents the time since the start of isolation (or another time-related variable, depending on the context of the study).
- The constant terms (24.01977 and 23.8279) represent the estimated viral load at time zero (the intercept).
- The coefficients (0.67608 and 0.6663) represent the rate of viral load decay over time, indicating the change in viral load per unit time.

Using Eq. (2), we estimated the human viral load decay from day one of occupancy for each patient over the course of their isolation period in the dorm. This estimate was then used as a random effect term to evaluate the correlation between environmental viral load data and census data, as explained in the previous section. Given that the coefficients of both equations are very similar (0.67608 and 0.6663), we elected to use Eq. (2) for our analysis, as it was derived from the same quarantine building under study.

Since building occupants did not undergo continued PCR testing after their initial positive PCR test and being admitted to the quarantine dormitory some assumptions regarding occupant viral load in the positive wing (north wing) were necessary.

Assumptions:

1. All occupant PCR tests were true positives.
2. All occupants in the positive wing were shedding detectable viral material.
3. Everyone in the positive wing has the potential to infect individuals presently negative for COVID-19.
4. Everyone in the positive wing will test negative after a period of time.
5. That period of time will vary from individual to individual.

## Results

### Experiment 1

Approximately 26% (46/179) of the air samples collected from the lobby air sampler were positive for SARS-CoV-2. The C_T_ values of the air samplers in the lobby followed the same general trend as the building census indicating a degree of negative correlation with the total building census (Pearson’s *r* = −0.223).

**Fig. 5:**
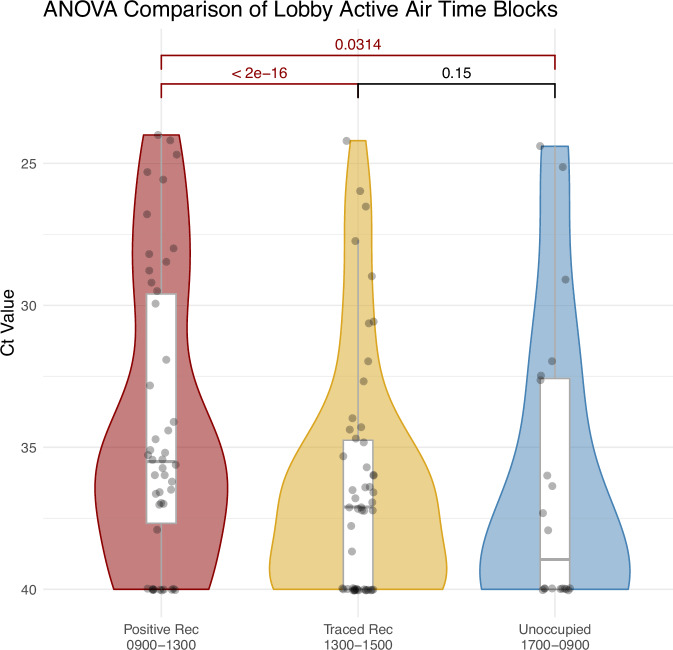
Violin plots with ANOVA test comparison of lobby air samples from consecutive daily time blocks. Whisker plots represent the median (Q2) bar and lower (Q1) and upper (Q3) quartiles in white with the remaining spread indicating the minimum and maximum values. Tests were performed with the log2 transformed Ct values to ensure data met a normal distribution. Note that lower C_T_ values indicate higher viral abundance and higher C_T_ values indicate lower viral abundance.This figure supports Experiment 1.

The C_T_ values for the lobby air samples collected during the “confirmed positive occupant” outdoor time and the two hours following (09:00–13:00) were statistically different from the “contact-traced” outdoor time period and the two hours following (13:00–17:00) (*p* > *2e−16*), and the overnight “unoccupied” 17:00–9:00 period (*p* = 0.0314). The lobby aerosol viral load from the “contact-traced” time period and the two hours following (13:00–17:00) did not vary significantly from the “unoccupied” time period (17:00–9:00) (Fig. 5). The two hours after the occupied periods were included due to virus lingering in the air following known aerosol viral load decay dynamics [31, 33].

### Experiment 2

**Fig. 6:**
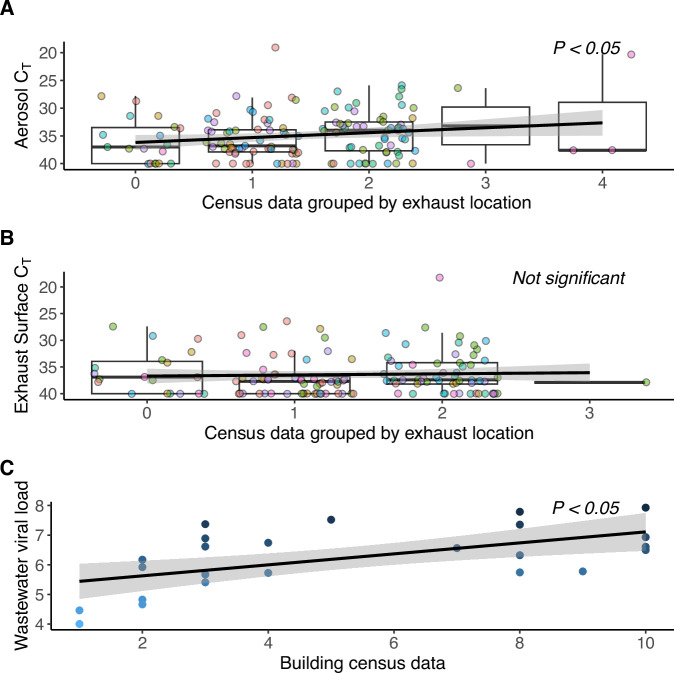
**A** Mixed linear regression model comparing rooftop exhaust aerosol sample C_T_ values to the census of confirmed positive occupants in rooms connected to the stack where the sample collected. **B** Mixed linear regression model comparing exhaust duct surfaces C_T_ values to the census of confirmed positive occupant census in rooms connected to the stack where the sample was collected. **C** Mixed linear regression model comparing log scale viral load in wastewater samples (log gc/L) to the confirmed positive occupant census and estimated human viral load. In panels **A** and **B** colored data points represent the different exhaust stacks and exhaust duct surfaces that the samples were collected from. Note that lower C_T_ values indicate higher viral abundance and higher C_T_ values indicate lower viral abundance. This figure supports Experiment 2.

Linear mixed-model analyses show statistically significant correlation between viral loads detected in air samples collected from rooftop exhaust air and census data and the associated estimated human viral load (Fig. 6). For these analyses, viral load measured at rooftop exhaust stacks that were connected to a cluster of rooms from different floors were considered as random effect terms in the regression model, interacting with average estimated human viral load for each cluster (*p* = 0.0413).

We performed a similar statistical model for surface swabs collected from the interior surface of each exhaust air duct; however, this correlation was not statistically significant (*p* = 0.75). We observed that the viral load detected in samples from high-touch surfaces throughout the building is correlated with the census data and estimated human viral load of the confirmed positive occupants in the building (*p* < 0.001).

Since the wastewater sampling data are indicative of the entire north wing of the building rather than a specific room or grouping of rooms, we performed a linear multi- regression model between the observed viral load (log_10_ gc/L) in wastewater samples and independent variables of census data and estimated human viral loads for confirmed positive occupants each day. For this analysis, the daily census data and estimated human viral load had a statistically significant correlation with wastewater (*p* = 0.0323).

Table 2 presents the number of positive and negative environmental samples organized around census status in the related room clusters.

**Table 2:** Pertains to Experiment 2.

	Rooftop exhaust aerosol samples	Exhaust duct surfaces	Wastewater	High-touch surfaces
Total samples	257	230	24	332
Positive samples & >0 positive students in rooms	55	30	21	153
Positive samples & 0 positive students in rooms	63	54	3	0
Negative samples & >0 positive students in rooms	59	78	0	179
Negative samples & 0 positive students in rooms	80	68	0	0
Putative false negative rate	23%	34%	0%	54%
Putative false positive rate	25%	23%	17%	0%
Rate of agreement with clinical case data	53%	43%	83%	46%

Number of positive or negative samples based upon census data in associated room clusters.

## Discussion

In this study, we demonstrated that wastewater sampling had the greatest apparent sensitivity and lowest putative false negative and putative false positive rates of the sampling modalities tested. Wastewater was also strongly correlated with positive clinical cases and therefore provided a capability of estimating the number of infected individuals in the building. However, wastewater surveillance provided less spatial resolution than other methods as it was unknown where in the building the infected individuals were. Additionally, wastewater sampling was limited to individuals using restrooms in the building, which may not accurately capture non-residents visiting a typical public building.

In contrast, due to the architectural and engineering design of the building, rooftop exhaust air sampling identified SARS-CoV-2 in indoor environments at a finer spatial resolution than wastewater sampling. Whereas wastewater sampling was able to detect signals at the scale of the north wing of the building, rooftop exhaust air sampling was able to detect signals at the resolution of room groups, connected by a single exhaust air stack. However, many building systems aggregate all exhaust air to a single or small number of air handler exhaust(s) and thus air sampling would be more akin to wastewater sampling in some buildings. Additionally, the inclusion of fresh air supply from hallways (which includes air from other rooms in the building) or open windows into exhaust air stacks servicing rooms may lead to higher putative false positive and putative negative surveillance results respectively. Importantly, these aerosol dynamics also drive changes in disease transmission risk in the indoor settings.

Surface sampling efforts had the greatest frequency of putative false negative results. Sampling the surfaces of rooftop exhaust ducts resulted in greater putative false negative results than aerosol sampling from those same rooftop exhaust ducts, likely due to increased exposure to the environment, including UV radiation and heat. However, sampling high-touch surfaces within the building resulted in the greatest putative false negative rate, likely due to the frequent use of disinfectants by custodial staff on these surfaces.

This study also demonstrated that long-period aerosol sampling can provide useful traces of infectious agents in valuable spatial resolution if short-period sampling in each individual space is not an available option. While large volume indoor room aerosol sampling (i.e., lobby air sampling) can provide near real-time information regarding the presence of viral particles in indoor air, these results may not align with the confirmed positive daily census data. Air samplers can also be easily moved to collect and monitor air in different spaces within a building if the building occupancy shifts spatially.

This study demonstrated that wastewater and aerosol sampling are effective and complementary methods for identifying and quantifying the presence of SARS-CoV-2 at the building and room-cluster scale. Both wastewater and aerosol sampling had low putative false positive rates, and legitimate arguments for 0% false positive detection. Together, these methods can be used in synergy to identify potential infectious outbreaks in a building (wastewater) and provide insights into its spread throughout the building at the zonal scale (aerosol sampling). Such a surveillance system would provide rapid and accurate data to enhance decision makers ability to create and enforce effective and timely policy actions.

### Experiment 1

When compared via a one-way ANOVA test, the C_T_ values from the “confirmed positive occupant” outdoor time period and the two hour period directly afterward (09:00–13:00, and 11:00–13:00) are statistically lower (higher abundance of detectable SARS-CoV-2 RNA) when compared to both the contact-traced outdoor time (13:00–17:00) and the overnight unoccupied” (17:00–09:00) time periods. The contact-traced occupant periods and all unoccupied time periods did not statistically differ. This indicates that when the confirmed positive occupants moved through the lobby (09:00–11:00), the amount of SARS-CoV-2 viral particles in lobby aerosols increased in detectable level. This increase in detectable SARS-CoV-2 RNA likely remained after the confirmed positive occupants were no longer moving through the lobby as identified in the samples collected during the two hours afterwards following typical viral decay dynamics [33]. Per ANSI/ASHRAE Standard 62.1-2022, this lobby is estimated to have approximately 0.5 air changes per hour in addition to infiltration from door operation. We observed that isolation dorm occupants tended to return to their rooms by moving through the lobby near the end of the outdoor time period (nearer to 11:00), and therefore a plausible explanation is that the SARS-CoV-2 RNA they shed remained detectable in room aerosols collected during the two-hour period afterwards (11:00–13:00). The aerosol samples collected from 13:00 to 17:00 had statistically lower viral load than those collected during the contact-traced occupant period and subsequent two-hour period thereafter, as well as during the overnight unoccupied period (17:00–09:00).

Strict adherences were applied to student recreation time for all building occupants. Occupants were free to leave the building once per day during their designated recreation period, resulting in a <1 min typical passage through the lobby when they left and an estimated <3 min passage (accounting for elevator wait time) again upon their return to their rooms. Moreover, when any person(s) entered or exited the lobby, additional outdoor air was introduced into the lobby through the opened door which can accelerate particle resuspension and mixing while concurrently diluting human aerosol particles in the lobby air. The results of this study, together with evidence that viable SARS-CoV-2 has been recovered from air samples [17, 18] and that human nasal positivity rates correlated with C_T_ values [42], we conclude that short duration (2–4 h) indoor air sampling is an effective method for detecting, and through surveillance, potentially limiting, human exposure to airborne infectious agents in large volume spaces.

### Experiment 2

Each method of environmental pathogen surveillance utilized in this experiment had strengths and limitations. For instance, the viral loads detected in rooftop exhaust aerosol samples (linear mixed effect model, *p* = 0.0413), high-touch surfaces (*p* < 0.001), and wastewater samples (multi-regression model, *p* = 0.0323) demonstrated significant correlations to the confirmed positive daily census counts and the estimated average human viral load. Relative concurrence of surveillance modality results may have varied due to environmental influences such as window operation impacting exhaust aerosol viral concentration, occupant use and cleaning practices impacting high-touch surface sampling, and frequency of toilet use and flushing impacting wastewater viral load. Furthermore, surveillance modality also varied by way of ease to setup (from easiest setup to most challenging: 1) high-touch surfaces, 2) rooftop exhaust, 3) wastewater), public visibility/intrusiveness of sampling locations (from least visible to most: 1) wastewater, 2) rooftop exhaust, 3) high-touch surfaces), ease of repeated access to sampling locations (from easiest to access to most challenging: 1) high-touch surfaces, 2) wastewater, 3) rooftop exhaust), duration required to collect samples (from least time to most 1) wastewater, 2) rooftop exhaust, 3) high-touch surfaces), and finally by spatial granularity of possible sampling locations (from granular to coarse:, 1) high-touch surfaces, 2) rooftop exhaust, 3) wastewater). Some of these factors likely also influenced the concurrence, putative false positive, and putative false negative rates of each surveillance modality with the viral load estimated from confirmed positive daily census data. Additional information about sampling configurations and confounding variables can be found in Supplementary Information Section 1.4.

Of the methods employed, wastewater sampling had the lowest putative false negative rate (0%) and the highest agreement with clinical surveillance rate (83%), a result that may not be surprising given it had the lowest spatial sampling granularity. This is in agreement with previous studies that have demonstrated the ability to detect individuals infected with SARS-CoV-2 at the building scale [45–47]. Additionally, wastewater SARS-CoV-2 concentrations have been shown to perform better than reported clinical cases in estimating the prevalence of COVID-19 in a community [48, 49].

Rooftop exhaust aerosol sampling had the second lowest putative false negative rate (23%) and second highest concurrence with confirmed positive occupants (53%). Building airflow dynamics, viral particle aerosol dilution and dispersion and room-to-room aerosol infiltration are all factors that could contribute to the putative false negative rate associated with rooftop aerosol sampling. For example, although occupants were confined to their rooms, the main door could be opened as many as five times per day (three times for meal delivery and two times for recreation breaks). Occupants also walked the hallways on their way to recreation time and thus were distributing viral load to the common area hallways and lobby, and these shared air spaces all of which exhaust through the individual occupant room bathroom exhaust stacks, possibly creating crossover effects. Finally, occupants were also able to open and close their windows (not monitored), which has been shown to decrease indoor aerosol viral load by approximately 50% [34], which may have diluted viral load available to the exhaust aerosol samples. Furthermore, the dorm rooms sampled had relatively low mechanical air changes per hour (ACH) with an average ACH of 0.56 (range 0.14–2.52) when the windows and bathroom doors were closed, with the exhaust air including increased volume of air from other parts of the building such as hallways and other rooms. When the windows and bathroom doors were opened, the average ACH increased to 0.74 (range 0.2–3.54) with the exhaust air including increased volumes of air from outside the building. In combination, these door room air exchange dynamics may have contributed to the (putative) false negative rate observed, but would similarly decrease the likelihood of disease transmission.

Surface sampling of exhaust air ducts had the next highest putative false negative rates at 34%. This is consistent with the observed results of non-significant correlation with daily census data for exhaust duct (*p* = 0.75), and a significantly low coefficient of determination for high-touch surfaces (*p* < 0.01, Adjusted r^2^ = 0.07). The exhaust duct surfaces were made of galvanized steel and contact-traced to the elements and therefore experienced temperature fluctuation consistent with an unprotected metal object placed in the sun on a rooftop. Furthermore, the duct surfaces were exposed to sunlight, both temperature and sunlight have been shown to inactivate SARS-CoV-2 [50, 51]. These factors could explain part of the (putative) false negative rate.

High-touch surface sampling had the highest putative false positive rate of all the surveillance methods examined (54%). This may be due in part to the high-touch surface cleaning protocols enacted by University of Oregon custodial staff. High-touch surfaces were disinfected, with either Clorox bleach spray or hand wipes, multiple times daily and likely led to the degradation and partial removal of detectable genetic material. However, as observed in the data, even with the frequent cleaning, it was possible to detect SARS-CoV-2 46% of the time when known infected individuals were present. This is in agreement with studies demonstrating the detection of SARS-CoV-2 on fully sanitized surfaces with the signal being detectable on material surfaces days after exposure to viral particles [43, 52]. However, the persistence of remnant RNA on surfaces can be an added barrier if the goal of the sampling regime is to track current infectious individuals in a building as samples could contain relic RNA from days beforehand. This could partially explain why the C_T_ values of the high-touch surface samples only correlated with building census at a low degree.

Interestingly, samples from wastewater, rooftop exhaust air and exhaust air duct surfaces all had similar putative false positive rates ranging from 17 to 25%. It is likely that this putative false positive rate is explained by two occupants who had tested negative for COVID-19 but had both been contact-traced to COVID-19 and had COVID-19 symptoms. These individuals were classified as “likely positive” by the University of Oregon and underwent isolation consistent with individuals who had tested positive. It is possible these individuals tested negative for COVID-19 before they had a detectable viral load and were in fact shedding virus afterwards. If these two “likely positive” cases were indeed positive for COVID-19 then the wastewater sampling correctly detected positive occupants in the north wing 100% of the time with a 0% false positive rate.

The rooftop exhaust aerosol sampling putative false positive rate also has a plausible explanation. In this case, the value for rooftop exhaust aerosol sampling also drops to 0% if you consider occupants within the entire wing (similar to wastewater sampling resolution) rather than the specific room cluster. Since infected occupants traversed common areas including the lobby and hallways where supply air is sourced before exhausting through grilles in the bathrooms of all rooms in the wing, it is possible that some of the putative false positives are actually correct detections of air originally sourced in the lobby and hallways exposed to larger groups of confirmed positive occupants.

### Practical applications

We suggest that these different sampling techniques can be used to guide building management techniques and mitigate illness spread. Employing 2–8 h lobby sampling could be used to determine viral load in shift workers and to guide human behavior changes (allowable occupant densities, masking requirements) and targeted human testing and contact-tracing when viral loads are rising. Longer period sampling such as 24 h wastewater, rooftop exhaust aerosol or lobby aerosol sampling could be used to guide building scale management techniques like increased ventilation, filtration, humidification or other decontamination techniques.

## Limitations

The study did not monitor individual isolation room window, door, or in-room occupant masking conditions, which would influence the composition of sampled exhaust air related to occupant C_T_ values. This study does not control for the potential that environmental sampling could have detected SARS-CoV-2 RNA shed by University of Oregon staff during the study period; however, research personnel underwent regular testing, were always masked (N95), and were advised to stay home if they felt ill or had COVID-19 symptoms.This study does not address whether detected SARS-CoV-2 RNA was viable and infectious, or non-viable RNA genetic material. Rooftop exhaust aerosol samples, rooftop exhaust duct surface samples and high-touch surface samples underwent RT-qPCR and were quantified by comparing C_T_ values. Wastewater samples were quantified via RT-ddPCR and were reported as gene copies per liter on a logarithmic scale (log gc/L). These are standard methods of lab processing for each respective sample type. Since the units differ, we do not compare the absolute values of wastewater log gc/L to surface and aerosol samples but instead examine trends in the different scales side by side to compare trends and fluctuations. The lobby air changes per hour were estimated using ANSI/ASHRAE standards since measuring the ACH was difficult due to the lobby having an open second floor connection to the second floor balcony and through there to second floor hallways.

## Supplementary information

Supplemental Information

## Data Availability

All data and scripts are available on GitHub.com here https://github.com/Andreas-Martinez/multimodal_surveillance.git.
