# Supplementary material for: Air, surface, and wastewater surveillance of SARS-CoV-2; a multimodal evaluation of COVID-19 detection in a built environment
Source: J Expo Sci Environ Epidemiol. 2025 Mar 1;35(4):672–82. doi: 10.1038/s41370-025-00757-3 (PMC12234356; doi:10.1038/s41370-025-00757-3)
Supplement: Supplementary file 1 — Supplemental Information [file 41370_2025_757_MOESM1_ESM.docx]

Supplemental Information

Air, surface, and wastewater surveillance of SARS-CoV-2; A multimodal comparison of COVID-19 detection in buildings

**Supplemental Content Summary**

This supplementary information section contains additional information about the methods of the study including laboratory analysis, student schedule, building occupation information and sampling setup. We also present an additional finding via Supplemental Figure 1 as well as a picture of the rooftop exhaust aerosol sampling setup.

**Supplemental Section 1: Methods**

1.1

*Laboratory Analysis*

For air and surface samples laboratory analyses were performed in a Type A2 biosafety cabinet (Purifier Logic+ Class II, LabConco Catalog #302420001). 400μL of each aerosol and surface sample was aliquoted and used as the input for RNA extraction using the Quick-DNA/RNA Viral Magbead kit (Zymo Research, Catalog #R2141) and protocol. 800 μL of lysis buffer and 20 μL magnetic beads were added to each well, the plate was sealed, and shaken for 10 minutes. The supernatant was removed, and the lysates were washed four times (1X with MagBead DNA/RNA Wash 1, 1X MagBead with DNA/RNA Wash 2, 2X with 100% ethanol). Nucleic acids were eluted into 50 μL nuclease-free water and stored at -80°C until downstream analysis. Successful RNA extraction was confirmed in each aerosol and surface sample through the addition of a 5 μL spike-in of *Escherichia coli* MS2 bacteriophage into each extraction well. Each extraction plate contained one extraction control well consisting of nuclease-free water in place of an aerosol and surface sample.

Aerosol and surface samples underwent quantitative reverse-transcription polymerase chain reaction (qRT-PCR) analysis using the TaqPath COVID-19 Combo Kit (Thermo Fisher Scientific, Catalog #A47814). This quadruplex qRT-PCR reaction targets the spike (S), nucleocapsid (N), and RNA-dependent RNA polymerase (RdRP/ORF1ab) genomic regions. The assay also targets the *Escherichia coli* MS2 bacteriophage as an internal control. The reaction mixtures included 5 μL TaqPath 1-Step Multiplex Mastermix without ROX (Thermo Fisher Scientific, Catalog #A28521), 9 μL nuclease-free water (Invitrogen, Catalog #4387936), 1 µL COVID-19 Real Time PCR Assay Multiplex Mix (Thermo Fisher Scientific, Catalog #A47814), and 5 µL of template RNA. Thermocycling was performed with the QuantStudio5 (Applied Biosystems) using the following cycling conditions: 25 °C for 2 minutes, 53 °C for 10 minutes, 95 °C for 2 minutes, and 40 cycles of 95 °C for 30 seconds and 60°C for 30 seconds. Aerosol and surface samples were considered positive if amplification was observed in two out of three genome targets with a cycle threshold (C_T_) value less than or equal to 35 [^46^](https://paperpile.com/c/3s5WEO/Idli). Each qRT-PCR plate contained a positive RNA control (SARS-CoV-2, Wuhan variant), a no-template control (nuclease-free water), and three extraction controls. All controls performed as expected.

The 24 hour wastewater composite samples, collected from the building’s north wing cleanout, were transported on ice to Oregon State University’s sample processing lab (BSL-2). Upon arrival, samples (8-70 mL, with mean of 37.7 mL + 15.4 mL) were filtered through a mixed cellulose ester membrane filter (Whatman catalog no. 7141-104, Buckinghamshire, UK). The filter was placed into a 2 mL centrifuge tube prefilled with DNA/RNA Shield (Zymo Research # R 1100-250) and homogenized via bead beating for 2 min. RNA was extracted from the DNA/RNA Shield lysate using the MagMAX™ Viral/Pathogen Nucleic Acid Isolation kit on a KingFisher Flex automated instrument (ThermoFisher Scientific, Waltham, MA). Appropriate controls (positive: SARS-CoV-2 N gene and human RNAse P RNA; negative: certified SARS-CoV-2-free human RNAse P RNA) and field blanks were included in extraction. The quantification of SARS-CoV-2 RNA was done through reverse transcriptase droplet digital PCR (RT-ddPCR; Bio-Rad ddPCR, QX200), using the Centers for Disease Control and Prevention (CDC) N1 and N2 assays with RNAse P as an internal control, as previously described in George et al (George AD, Kaya D, Layton BA, Bailey K, Mansell S, Kelly C, et al. Impact of Sampling Type, Frequency, and Scale of the Collection System on SARS-CoV-2 Quantification Fidelity. Environ Sci Technol Lett. 2022 Feb 8;9(2):160–5.)

1.2

*Experiment 1 Student Schedule*

The confirmed positive students and contact-traced students had two hours of recreation time per day. Students who had received a positive COVID-19 test were housed on floors 1-4, students who were contact-traced were housed on floors 5-7. The 1st-4th floors and the 5th-7th floors were each accessed by their own respective elevators or by a communal stairway.

The recreation times were broken into two discrete time blocks, 0900-1100 for confirmed positive students and 1300-1500 for the contact-traced students. During the recreation times the students could exit their rooms and travel directly through the building as long as they were either leaving the building to recreate outside or returning straight to their rooms, whether the students used the stairway or the elevators they entered and exited through the lobby. The most time that any student spent in the lobby was when waiting for the elevator to arrive to return to their rooms. Aerosol samples from the lobby samplers were collected approximately every two hours in consistent and consecutive time blocks that were selected to capture two-hour time periods when students were on their breaks and moving through the lobby as well as the unoccupied periods before and after the breaks (Table 1). The aerosol samplers were also run overnight and collected in the morning for a longer duration (Table 1).

1.3

*Experiment 2 Occupation*

Students who had been contact-traced to an exposure event but had not received a positive COVID-19 test were admitted to the south wing of the building so that the sensitivity of the environmental techniques could be tested against a known quantity of confirmed positive COVID-19 cases in the north wing only. By separating the positive cases into the north wing, it was possible to spatially separate the results collected from the rest of the building and the rest of the buildings occupants. Daily building, wing and room census was tracked for each day of the study period. Samples were collected daily for 29 consecutive days with slight variation in the launch and end date of the wastewater and surface samples due to deployment challenges and equipment availability restrictions.

During the 29-day Experiment 2 study period, the population of confirmed positive individuals in the north wing ranged from 1-15 during the study. Sample collection days 1-6 took place during Spring Break while many students were traveling or visiting home and continued for several weeks after Spring Break ended and students returned back to campus. Therefore, the populations in both the whole building and the north wing started low, increased and then decreased in a rough bell curve before sharply increasing in the last 5-7 days of the study (Fig. 3a).

1.4

*Experiment 2 Sampling Setups*

*High-Touch surface swabs*

High-touch surfaces inside the building were identified and swabbed once every 24 hours. A total of 6 surfaces were swabbed (approximate sampling area = 600 cm^2^ per sample) with flocked nylon fiber swabs (Typenex Medical LLC, Catalog #SW0202) pre-moistened with DNA/RNA Shield (Zymo Research, Catalog #R1100). The surfaces swabbed were the lobby west entrance door handle exterior, lobby west entrance door handle interior, lobby elevator call button, lobby stairway door handle, isolation floor elevator railing, isolation floor isolation floor elevator buttons, isolation floor elevator call button, isolation floor hallway door handle, isolation floor stairway door handle. High touch surfaces were swabbed once every 24-hour period for 26 consecutive days. Swabs were then placed into a 1 mL of the DNA/RNA Shield for transport to an on campus BSL-2 laboratory for further processing and analysis and storage.

*Wastewater Setup 1*

Two different sampling layouts were attempted before an effective sampling technique was reached on the second setup. All data included in this study was collected from Setup #2. No data used in the study was collected from wastewater setup 1, we present this setup to share the challenges we encountered when attempting to sample through a cleanout drain.

The first wastewater sampler (Manning Environmental Inc. Model PSB) was deployed into the cleanout pipe and set to collect 50 mL of sample every 15 minutes for 24 hours. Researchers then flushed toilet paper down toilets throughout the building to fortify the dam to allow a pool to build and submerge the collection diffuser. In order to keep the wastewater samples cold the researchers placed the sampler inside a sealable case and filled the case with frozen ice packs which were replaced every 12 hours. Due to low census in the north wing of the building, researchers flushed toilets on several days to ensure there was enough water flow through the north cleanout pipe to be captured by the wastewater autosampler. Even with researchers flushing toilets, sample amounts were intermittent and often below the 50 mL volume necessary for laboratory analysis. The sampling amounts and times were changed to 25 mL every 5 minutes and 10 mL every 3 minutes to try and increase the sample volume to the 50 mL needed. The sampling tube and strainer set up was adjusted 3 times and the snake camera was threaded into the cleanout pipe which determined that the sampling strainer had washed out of the sewer fluid onto the side of the pipe likely leading to poor capture volumes. This initial setup was abandoned due to insufficient sample amount and an inability to keep samples properly refrigerated during the 12 hours between ice pack changes.

*Wastewater Setup 2*

All data presented in this study was collect using setup #2.

Researchers switched wastewater samplers to an ISCO 3700C autosampler whose sampling strainer was inserted into the north cleanout pipe. This cleanout is a 4-inch diameter cleanout of an 8-inch diameter pipe located in the building's maintenance shop on the ground floor. Additionally, an inflatable IV bag was inserted into the cleanout pipe and was inflated to create a dam downstream of the sampling strainer. The autosampler was set to extract 100 mL every 10 minutes for a 24-hour period with the collected samples being kept on ice. Sample amounts varied daily, likely due to normal fluctuations in the amount of water that was used in rooms that feed into the north cleanout pipe as well as the tendency for the in-pipe dam to shift over time and the possibility that the sampling sampler was not always submerged or temporarily clogged. Wastewater sample amounts utilizing this second setup were much higher than in the first sampling setup and fell within the 50 mL-10 L range, often well above the 50 mL required for analysis. Sample amounts greater than 50 mL were well mixed prior to aliquoting 50 mL for analyses. Samples were stored on ice and transported to Oregon State University where laboratory analysis was conducted by the Radniecki Research Group at Oregon State University (Corvallis, Oregon).

*Exhaust Duct Surface Swabs*

Exhaust duct surfaces took the research team less than a day to identify ideal sample sites where a high degree of indoor air impacted the exhaust duct surface and to gather and deploy the necessary sampling equipment, sample collection took ~15 minutes each day. Sampling these surfaces was completely unobtrusive to the building occupants as sampling took place exclusively on the locked roof of the building.

As with the exhaust duct surfaces the high-touch surface sampling has been a common method of detecting SARS-CoV-2 and assessing cleanliness and cleaning practices in buildings, particularly in hospitals, long-term care facilities, food production facilities, offices and other facilities. It took the research team less than 24 hours to identify ideal high-touch and communally located sample sites and to gather and deploy the necessary sampling equipment, sample collection took ~15 minutes each day. High-touch surface sampling is very visible to building occupants who may be concerned to see staff in PPE collecting biological samples from the building.

*Institutional Review and Approval:*

All biological sample collection and management protocols were approved by the Advarra Institutional Biosafety Committee (IBC)(Protocol #PROTO202000132). Advarra IBC is approved by the University of Oregon as an external IBC and is registered with the National Institute of Health (NIH).

**Supplemental Section 2: Experiment 2 Figures and Tables**

| Supplemental Table 1 - Rooftop Exhaust Airflow Rates | |
| --- | --- |
| **Exhaust Fan #** | **Stack Exhaust Airflow (L/m)** |
| 26 | 5493.46 |
| 27 | 2831.68 |
| 28 | 4842.17 |
| 29 | 8098.60 |
| 30 | 6314.65 |
| 31 | 6796.03 |
| 32 | 8381.77 |
| 33 | 6739.40 |
| 34 | 5663.36 |
| 37 | 6286.33 |
| 38 | 3341.38 |
| 39 | 4728.91 |
| 40 | 5833.26 |
| Average | 5796.17 |
| Minimum | 2831.68 |
| Max | 8381.77 |
| Sum | 75351.00 |

Supplemental Table 1: Measured airflow liter per minute (L/m) exhaust flow rate for each rooftop exhaust fan sampled in Experiment 2. Measurements were recorded over 1 minute and averaged for each air stack.


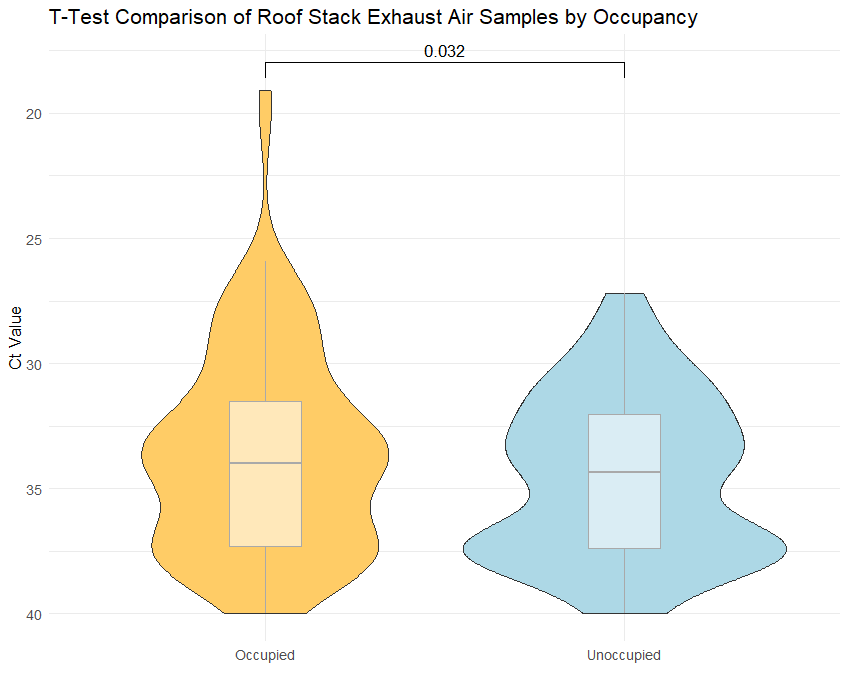
 Supplemental Fig. 1: Daily C_T_ values of unoccupied vs occupied stacks compared by one-tailed Welch’s T-test. C_T_ values of occupied stacks are significantly lower (*p* = 0.032). Multiple positive aerosol samples were collected from air of stacks that had no occupied rooms attached to them during the sampling period. This is likely due to SARS-CoV-2 RNA making its way from areas of the building occupied by COVID-19 positive individuals into the exhaust vents through room-to-room infiltration.

**Site image**


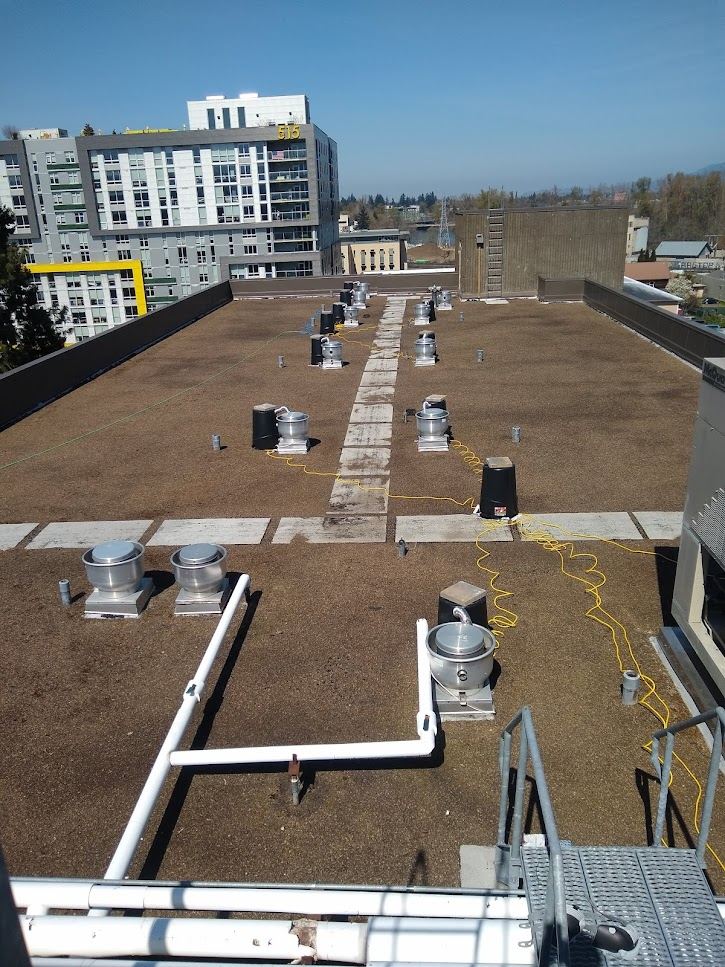


Supplemental Image 1: Image of rooftop air sampler setup. Air samplers were placed under inverted plastic trash cans and weighed down with cinder blocks to keep the samplers dry.
